# A Bridge Between Oral Hygiene and Critical Care: Reviewing a Variety of Oral Hygiene Interventions on Critically Ill Patients to Prevent Ventilator-Associated Pneumonia (VAP)

**DOI:** 10.7759/cureus.114164

**Published:** 2026-08-08

**Authors:** Subramanian Chokkalingam

**Affiliations:** 1 Department of Intensive Care Medicine, Health Education North West, Liverpool, GBR

**Keywords:** mechanical ventilation, microaspiration, nosocomial infection, oral hygiene maintenance, oral microbiome dysbiosis, ventilator-associated pneumonia

## Abstract

Ventilator-associated pneumonia (VAP) is one of the common causes of severe morbidity and mortality in critically ill patients who are under mechanical ventilation for more than 48 hours. VAP is multifactorial, and poor oral hygiene could be one of the contributing factors for the development of VAP. The main reason is the aspiration of oropharyngeal secretions containing pathogenic microorganisms, eventually leading to colonisation of virulent oral microorganisms in the respiratory tract, which could potentially increase the risk of development of pneumonia. In spite of having better clinical knowledge, greater experience, and advanced technologies, intensivists are still facing challenges in preventing VAP in mechanically ventilated patients. There are various evidence-based strategies employed to maintain better oral health in critically ill patients to prevent the incidence of VAP.

This review aims to discuss the different interventions used to maintain oral health in mechanically ventilated patients.

## Introduction and background

A parenchymal lung infection that occurs in patients who are hospitalised for 48 hours or more is known as nosocomial pneumonia [1]. Critically ill patients who receive mechanical ventilation are more prone to this infection. Numerous studies have shown that ventilator-associated pneumonia (VAP) is the most common nosocomial infection acquired by patients who are admitted to the Intensive Care Unit (ICU) [2,3]. 

Historically, surveillance within the CDC National Healthcare Safety Network (NHSN) focused on ventilator-associated pneumonia (VAP). However, because traditional surveillance definitions relied on subjective clinical and radiographic criteria with limited reproducibility, the CDC convened a multidisciplinary Working Group in 2011 to develop a more objective surveillance framework. Since 2013, the NHSN has adopted the Ventilator-Associated Event (VAE) algorithm, which classifies events into three hierarchical tiers [4]: (1) Ventilator-Associated Condition (VAC): Defined by sustained worsening oxygenation following a period of ventilator stability or improvement; (2) Infection-related Ventilator-Associated Complication (IVAC): Adds evidence of systemic infection or inflammation and new antimicrobial therapy; and (3) Possible Ventilator-Associated Pneumonia (PVAP): Incorporates microbiological evidence suggestive of pulmonary infection.

The oral cavity comprises a diverse range of microorganisms that exist in a symbiotic relationship and protect against the colonisation of pathogenic microorganisms. The existing natural symbiosis is altered when the healthy microbial balance is disrupted due to the systemic condition of the patient, underlying pathology, or the medical devices and equipment inserted into the patient’s airway, compromising the innate host defence by impairing protective airway reflexes. The altered symbiosis results in a pathological state called oral dysbiosis [5,6]. This dysbiotic environment harbours inflammatory mediators and respiratory pathogens in the diseased oral cavity, thereby leading to an increased risk of respiratory illness such as pneumonia when the contents of the oral cavity are aspirated during mechanical ventilation [7,8]. Therefore, many strategies have been implemented to maintain oral hygiene in critically ill patients for the prevention of VAP.

The objective of this narrative review is to highlight the various oral hygiene interventions that are practised in the critical care setting to prevent ventilator-associated pneumonia in patients who are admitted to the ICU.

## Review

Search methodology

Various databases, including PubMed, Scopus, and Google Scholar, were employed to achieve a comprehensive literature search to write this narrative review. A combination of search terms used was ‘ventilator-associated pneumonia’, ‘critically ill’, ‘oral hygiene’, ‘microaspiration’, ‘intensive care units’ and ‘dysbiosis’. Articles published as early as the 1990s were referred to in order to understand the clear history and background, and more recent articles published up to 2026 were reviewed to expand our knowledge on the recent advances and future trends. Peer-reviewed original articles, randomised controlled trials, systematic reviews and meta-analyses were included in the search methodology to provide a contemporary overview of the role of oral hygiene interventions in preventing ventilator-associated pneumonia in critically ill patients. As the article is a narrative review, the literature was identified and synthesised qualitatively rather than through a formal systematic study-selection process or quantitative meta-analysis.

Ventilator-associated pneumonia (VAP)

An infection that develops in the lung parenchyma in patients who are critically ill and mechanically ventilated in the ICU for more than 48 hours is called ventilator-associated pneumonia (VAP) [9].

The VAE algorithm was developed for epidemiological surveillance, benchmarking, and quality improvement rather than bedside clinical diagnosis. Consequently, these surveillance definitions should not be considered interchangeable with the clinical diagnosis of VAP, which remains based on the integration of clinical findings, radiographic evidence, and microbiological data to guide patient management [4].

The diagnosis of VAP is based on the presence of a new or progressive pulmonary infiltrate on chest imaging along with clinical manifestations, including fever or hypothermia, leukocytosis or leukopenia, purulent tracheobronchial secretions, and impaired oxygenation. Before these findings can be attributed to VAP, diagnoses like pulmonary oedema, atelectasis, acute respiratory distress syndrome, pulmonary haemorrhage, or pulmonary embolism should be excluded. Microbiological analysis of respiratory specimens, including endotracheal aspirates, bronchoalveolar lavage fluid, and protected specimen brush samples, provides supportive diagnostic evidence and aids in the selection of antimicrobial therapy [10].

There are various risk factors associated with the incidence of VAP (Table 1). Torres A et al. (1990) [2] found that more than one tracheal intubation during mechanical ventilation, patients aged more than 60 years, increased duration of ventilation for more than 3 days, and aspiration of gastric contents could precipitate the occurrence of VAP. The presence of a nasogastric tube, increased infections by antibiotic-resistant pathogens, other systemic illnesses such as chronic obstructive pulmonary disease (COPD), hypoalbuminaemia, uraemia and their severity were associated with the development of VAP [3]. Seasonal changes, bed position of the patient, and transfusion of more than four units of blood were also found to be risk factors for the incidence of VAP [11].

**Table 1:** Various risk factors associated with the incidence of VAP VAP: ventilator-associated pneumonia

Hospital-associated factors	Host factors
Tracheal intubation	Age more than 60 years
Increased duration of ventilation	Severity of illness
Usage of nasogastric tube	Immune status
Sedation	Supine position
Transport out of ICU	Volume aspirated
More than 4 units of blood products	Other infections/systemic illness

**Figure 1:**
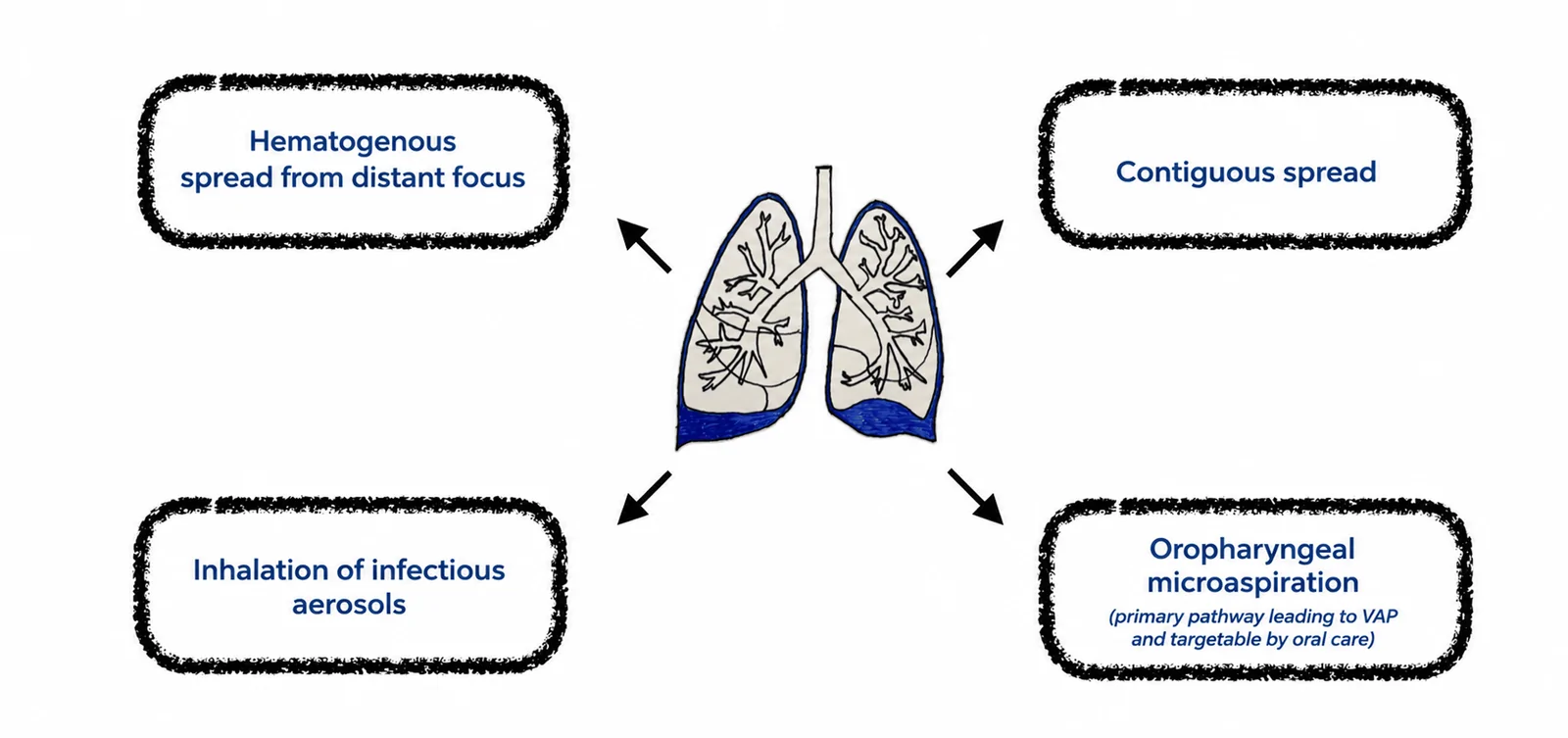
Schematic representation showing the routes of incidence of ventilator-associated pneumonia Created by the author using Microsoft PowerPoint (Microsoft Corporation, Redmond, USA).

There are four major routes by which the spread of VAP occurs (Figure 1). It includes spread from a distant focus through the blood, spread from an adjacent site to the lungs, inhalation of pathogens into the lungs, and aspiration of oral contents containing pathogenic microorganisms. Out of these four routes, aspiration of oral pathogens and colonisation of Gram-positive and Gram-negative organisms in the lung parenchymal tissue is considered to be the major route of spread of VAP [1].

The infiltration of these organisms in the upper respiratory tract and eventual spreading of infection into the lungs is usually prevented by innate host defence. But patients who are ventilated in the ICU have an altered or reduced immune response due to their underlying disease and/or because of the equipment and devices that are inserted into them [9]. For this very reason, VAP is considered to be one of the most common infections in patients who are critically ill.

Various strategies used in the maintenance of oral hygiene

It is difficult to maintain good oral hygiene in critically ill patients due to the connected equipment and ventilators. This contributes to changes in the normal balanced environment of the oral cavity, leading to the proliferation of pathogenic microorganisms and a state called dysbiosis [12].

Numerous studies suggest that the microaspiration of these pathogens in intubated patients is the major etiology for the development of VAP [1].

A variety of strategies have been implemented in the maintenance of the oral cavity in critically ill patients to prevent the incidence of VAP. These strategies are discussed below.

Mechanical plaque removal

Toothbrushing

Mechanical disruption of plaque using a toothbrush was found to be more effective in reducing oral pathogens. A meta-analysis by Ehrenzeller S and Klompas M (2024) evaluated the effectiveness of toothbrushing in the prevention of VAP and found that daily toothbrushing reduced the rates of pneumonia and ICU mortality [13]. Although the effectiveness of toothbrushing was influenced by the rigour, intensity and frequency of toothbrushing on a daily basis, the overall result showed a consistent reduction in the incidence of pneumonia and decreased the length of hospital stay.

Other Mechanical Aids

A survey by Soh KL et al. (2012) highlighted the importance of using mechanical aids such as forceps with cotton, gauze, and spatulas for maintaining good oral hygiene among ventilated patients [14].

Chemical agents

Chlorhexidine

Chlorhexidine is a polycationic bisbiguanide that acts as an effective antiseptic used in different concentrations and forms. Many healthcare settings, including intensive care units, use chlorhexidine widely to act against various Gram-positive and Gram-negative bacteria and to prevent VAP. However, evidence regarding its effectiveness remains heterogeneous. Although clinical studies suggest that chlorhexidine in paste form at a concentration of 2% reduces the incidence of VAP [15], these findings have not been consistently replicated in all ICU populations or have shown improvements in patient-centred outcomes such as mortality, duration of mechanical ventilation, or ICU length of stay. Therefore, a careful interpretation of its benefits should be made, and chlorhexidine should be considered as one of the comprehensive oral care strategies rather than a universally effective intervention for VAP prevention.

Side Effects: The major side effects reported with the use of chlorhexidine mouthwash and gels are altered taste, xerostomia, changes in tongue colour, plaque buildup, and discolouration of the teeth over time. Other less common side effects include parotid gland swelling, pain in the tongue, and desquamation of the mucosal lining [16]. These side effects are usually minor and resolve upon discontinuation. Severe allergic reactions were also reported due to chlorhexidine usage [17].

Povidone-iodine

Povidone-iodine is often seen as one of the best antimicrobial agents for reducing the chance of respiratory infections, including VAP. A systematic review by Emami Zeydi A et al. (2023) suggested that povidone-iodine is effective against a wide range of bacteria, including those commonly connected with VAP, and also reduces the length of hospital stay in critically ill patients [18]. Tsuda S et al. (2020) concluded that using 10% povidone-iodine for cleaning and irrigating the mouth instead of 0.12% chlorhexidine helps to control bacterial growth in the oropharynx of patients on ventilators without affecting the homeostasis of the oral bacteria [19].

Saline

Normal saline has been widely used as a cleansing or irrigating agent that facilitates the mechanical removal of oral debris and secretions. A network analysis by He Q et al. (2025) suggested that saline solution is a promising alternative as an antibacterial mouthwash that showed a significant reduction in mortality rates in ventilated patients admitted to the ICU [20].

Sodium Bicarbonate

Sodium bicarbonate, also known as baking soda, is a cleansing agent that can dissolve mucus and remove debris from tooth surfaces. It is considered to be a safer option to be used as a mouthwash and as part of toothpaste and chewing gum, as it is not too harsh on the teeth and can kill bacteria. This helps to reduce the number of bacteria in the oral cavity, thereby reducing the formation of dental plaque [21]. Therefore, sodium bicarbonate has considerable potential to help with oral hygiene in the ICU to prevent VAP. A randomised controlled trial by Loha S et al. (2022) showed that the usage of 0.9% sodium bicarbonate along with chlorhexidine as an oral rinse was found to reduce the incidence of VAP when compared to the usage of chlorhexidine alone [22].

Octenidine

Octenidine is an anti-plaque agent that has a broad range of antibacterial activity against Gram-positive and Gram-negative bacteria. A comparative study by Koburger T et al. (2010) evaluated the antibacterial efficacy of octenidine and found that it has superior characteristics as an antiseptic, when compared to chlorhexidine, povidone-iodine and triclosan [23]. An in vitro study by Zumtobel M et al. (2009) compared the antibacterial efficacy of octenidine dihydrochloride (OCT) coated polymer tracheostomy tubes with conventional tubes. It was found that OCT-coated tubes inhibit the colonisation of certain bacterial species that could potentially reduce the risk of developing VAP in ventilated patients [24].

Triclosan

Triclosan is a broad-spectrum antimicrobial agent used in the oral hygiene maintenance of intubated patients to minimise the incidence of VAP. It is used for effective plaque control due to its antibacterial nature [25]. Although numerous studies have suggested the antibacterial effects of triclosan, further research needs to be undertaken to prove its efficacy in reducing the risk of VAP in critically ill patients.

Furacilin

Furacilin, also known as nitrofural, is an antibacterial agent used as a mouth rinse and for topical wound debridement. A meta-analysis by Sankaran SP and Stephen Sonis (2021) concluded that furacilin, either with or without toothbrushing, acts as a potent antiseptic mouth rinse that best prevents VAP in critically ill patients [26].

Listerine®

Listerine® (Kenvue Brands LLC, Summit, USA), a phenolic compound, is an essential oil-containing mouthwash used in routine oral care. It was found to effectively reduce dental plaque due to its germicidal and anti-plaque properties [27]. A meta-analysis by Li S et al. (2024) found that Listerine mouthwash had superior benefits in reducing bacterial counts on mechanically ventilated patients in the ICU when compared to saline [28].

Side Effects: One of the components of Listerine® is thymol, which has cytotoxic effects. Listerine contains alcohol. This ethanol is a recognised carcinogen that is metabolised into acetaldehyde by the oral bacteria, leading to DNA damage in host cells [29]. This has led to theoretical concerns regarding the long-term use of alcohol-containing mouthwashes. However, there is no strong clinical evidence that suggests the potential carcinogenic effect of Listerine® on human cells. Therefore, this should be considered as a biological hypothesis rather than evidence of proven clinical harm.

Polyhexanide

Polyhexanide is a cationic biguanide polymer that binds to negatively charged phospholipids in the bacterial cell wall and destabilises the cell membrane, causing bactericidal action [30]. An in vitro study by Kollmuss M et al. (2021) found that 0.15% polyhexanide mouth rinse acts as a potent disinfectant against oral pathogens and significantly reduces CFU/ml, suggesting anti-biofilm properties [31]. A randomised controlled trial by Fernández-García C et al. (2016) studied the efficacy of polyhexanide as an oral care measure in tracheotomised patients. Although no significant difference was observed in the reduction of local infections between the usage of polyhexanide and povidone-iodine, it remains a potential option that polyhexanide can be used as an oral care measure to prevent VAP in ICU patients [32].

Hydrogen Peroxide

Hydrogen peroxide, also known as H_2_O_2_, is a clear, colourless, and odourless liquid. It has been used in dentistry since the early 1900s, either on its own or together with other salts. The FDA approved the use of 3% H_2_O_2_ as a temporary way to clean the mouth, especially for debridement [33]. Nobahar M et al. (2016) found that people who received 3% H_2_O_2_ mouthwash twice a day had a reduced incidence of VAP when compared to those who used saline [34].

Side Effects: H_2_O_2_ has some genotoxic effects in bacterial and mammalian cells. Long-term use of 3% H_2_O_2_ was associated with the development of pre-cancerous growths and tumours [35].

Potassium Permanganate

Potassium permanganate acts as a disinfectant that has astringent properties. Due to the strong oxidising nature of potassium permanganate, it was historically used as an antiseptic in the management of mucocutaneous infections. It neutralises toxins and pathogens due to its oxidising properties. Meidani M et al. (2018) found that 0.01% potassium permanganate as an oropharyngeal irrigation is as effective as 0.2% chlorhexidine in reducing VAP in ventilated patients [36]. Recently, the usage of potassium permanganate has become limited because of safety concerns. 

Side Effects: The oxidising nature of potassium permanganate causes skin irritation and chemical burns in the oral mucosa if not properly diluted. If used on mouth ulcers, it may stain the ulcer purple, making it difficult to remove.

Ozonated Water

Ozonated water is used as a pre-procedural rinse in many dental procedures to disinfect the oral cavity. It is used along with specialised oral irrigators for subgingival irrigation. Reactive hydroxyl radicals with oxidising properties are generated when ozonated water rapidly decomposes. These free radicals exert bactericidal effects [37]. A randomised controlled trial compared chlorhexidine and ozonated water as mouthwash solutions to assess the risk of development of VAP. The study concluded that the incidence of VAP was 45.9% in the chlorhexidine group when compared to a 25% incidence in the ozonated water group, suggesting better results with ozonated water when compared to chlorhexidine [38].

Nanosil

Nanosil mouthwash is composed of hydrogen peroxide and silver nanoparticles. The proliferation of bacteria is prevented due to the bactericidal action of hydrogen peroxide [39]. A randomised controlled trial by Khaky B et al. (2018) compared the efficacy of Nanosil mouthwash with 0.12% chlorhexidine in the prevention of VAP. The diagnosis of VAP was carried out with a version of the Modified Clinical Pulmonary Infection Scale (MCPIS). After five days of intervention, the Nanosil mouthwash group showed a statistically significant reduction in MCPIS scores when compared to the chlorhexidine group [40].

Miswak

Miswak is the stem of *Salvadora persica*, used as a herbal mouthwash due to its antibacterial properties. Li S et al. (2024) conducted a systematic review to compare the efficacy of herbal mouthwash, including miswak mouthwash, with chlorhexidine and found that the incidence of VAP was much less with the usage of miswak than with chlorhexidine [28].

Miscellaneous

Maintenance of Salivary Flow

Salivary flow rates should be maintained to enhance their self-cleansing effects and to gain immunological benefits that neutralise the virulence of oral pathogens, thereby reducing the potential Gram-negative bacterial colonisation. Increased salivary flow in ventilated patients also reduces the chances of xerostomia-induced mucositis [41].

Biotène® Oralbalance

Biotène® Oralbalance gel (Haleon, Surrey, UK) is a moisturising gel used often as a substitute for saliva to prevent xerostomia and mucositis. It contains enzymes like lactoperoxidase, lysozyme and lactoferrin which have antibacterial action [42]. Stefanescu BM et al. (2013) studied the efficacy of Biotène® Oralbalance in gel form as timed oral care in critically ill neonates and found that it reduced the incidence of VAP in ventilated infants when compared to the usage of sterile water rinse [43].

Multi-Modal VAP Prevention Strategies

Saline Instillation: Caruso P et al. (2009) studied the association of VAP and isotonic saline instillation before tracheal suctioning and found that there was a significant reduction in the incidence of VAP in patients who received saline instillation when compared to those who did not [44].

Subglottic Suctioning: Pathogenic organisms may originate from the subglottic secretions that could potentially lead to VAP. Evidence from Caroff DA et al. (2016) concluded that subglottic secretion drainage has a higher impact in preventing VAP when compared to no subglottic secretion drainage [45].

Head Elevation: Patient positioning is crucial in the prevention of VAP in mechanically ventilated patients. Elevating the head of the bed (HOB) has been found to be one of the safest and most effective ways to minimise the bacterial culture in endotracheal aspirate. A study by Guner CK and Kutluturkan S (2022) found that a semi-recumbent position of around 45° effectively prevented VAP when compared to less than 30° or 35° positions [46].

Future trends

A thorough knowledge of the oral hygiene status of patients admitted to the ICU helps in precise delivery of oral care and reduces the incidence of VAP in critically ill patients. This can be achieved by adopting the use of emerging AI diagnostic systems. AI diagnostic systems can detect oral diseases and assess periodontal status, enhancing the efficiency and accuracy of the diagnosis [47]. Butnaru OM et al. (2025) aimed to study the efficacy of AI as a complementary tool or if its limitations outweigh the advantages in the diagnostic field. The study compared Planmeca Romexis 6.4.7 software (Planmeca, Helsinki, Finland), an AI diagnostic system, with the diagnoses of general dentists and senior specialists. The results of the AI system were more consistent with the diagnoses made by senior specialists than with those of general dentists [48].

Contaminated toothbrushes, when used on ventilated patients, increase the risk of infection. This can be reduced by the use of single-use oral care sets. A study by Unahalekhaka A et al. (2025) evaluated the efficacy of single-use oral care sets incorporating a disposable toothbrush with integrated suction to remove water and saliva while brushing in reducing the risk of VAP. It was found that the usage of disposable toothbrushes with integrated suction reduced the incidence of VAP significantly [49].

In spite of having various adverse effects, chlorhexidine remains the primary option in the maintenance of oral care. Future trends focus on developing reliable technology that uses nanomaterials for targeted delivery of active agents [40]. The incorporation of nanotechnology in endotracheal tube coatings also provides antibacterial action that helps prevent the incidence of VAP. Silver-based nano-coatings on endotracheal tubes reduce the incidence of VAP in ICU settings by preventing bacterial adhesion to the tube and thereby breaking the bacterial cycle of colonisation and infection [50].

To provide a better and easier understanding of the various oral care interventions that are used to prevent VAP, Table 2 summarises the study design and key findings of each of the articles that have been referenced in this review. 

**Table 2:** Summary of the study design, key findings, population, primary outcome, limitations, and level of evidence of the reference articles included in this review CDC/NHSN: U.S. Centers for Disease Control and Prevention National Healthcare Safety Network

Author (year)	Study design	Key findings	Population	Primary outcome	Limitation	Level of evidence
Rello J et al. (2003) [1]	Narrative Review	Reviewed the microbiology profile, diagnosis, prevention, and management of nosocomial pneumonia.	ICU patients with nosocomial pneumonia	Prevention and management of nosocomial pneumonia	Narrative review	Low
Torres A et al. (1990) [2]	Prospective cohort study	Found that prolonged mechanical ventilation, severity of illness, and reintubation are risk factors for nosocomial pneumonia.	Mechanically ventilated ICU patients	Nosocomial pneumonia	Observational design	Moderate
Alp E et al. (2004) [3]	Prospective observational study	Nosocomial pneumonia was associated with increased ICU mortality; prolonged ventilation and invasive devices were risk factors.	ICU patients	Incidence, mortality, and risk factors for nosocomial pneumonia	Single observational cohort	Moderate
CDC/NHSN manual (2025) [4]	National surveillance guidance/manual describing the CDC/NHSN Ventilator-Associated Event (VAE) surveillance algorithm.	VAE algorithm defines Ventilator-Associated Condition (VAC), Infection-related Ventilator-Associated Complication (IVAC), and Possible Ventilator-Associated Pneumonia (PVAP).	Hospitalised patients with mechanical ventilation	NA	VAE algorithm was designed for surveillance rather than clinical diagnosis	NA
Dong J et al. (2022) [5]	Narrative review	Relates oral microbiome dysbiosis with respiratory diseases, emphasises oral hygiene as a potential preventive strategy.	Human and experimental studies	Relationship between oral dysbiosis and respiratory infection	Narrative synthesis	Low
Rogers et al. (2024) [6]	Multicenter cohort study	Explained that salivary microbiome differs in patients with acute lower respiratory tract infections, suggesting compromised immune status.	Patients with acute lower respiratory tract infection	Microbiome differences associated with respiratory infection	Observational	Moderate
Liu X et al. (2025) [7]	Narrative review	Reviewed the relationship between oral microbiota and respiratory diseases.	Human studies	Current evidence on oral-respiratory interactions	Narrative review	Low
Mehtonen IT et al. (2019) [8]	Population-based cohort study	Demonstrated that host inflammatory mediators increased risk of lower respiratory tract infections, supporting the importance of oral health in preventing respiratory disease.	General population	Lower respiratory tract infection	Non-ICU population; observational	Moderate
Kollef MH (1993) [9]	Prospective multivariate analysis	Identified risk factors for ventilator-associated pneumonia, including duration of mechanical ventilation and severity of illness.	Mechanically ventilated ICU patients	Ventilator-associated pneumonia	Observational design	Moderate
Kalil AC et al. (2016) [10]	Evidence-based clinical practice guideline developed by the Infectious Diseases Society of America (IDSA) and the American Thoracic Society (ATS)	VAP remains a clinical diagnosis integrating radiographic, clinical, and microbiological findings rather than a single diagnostic test.	Patients with hospital-acquired pneumonia (HAP) and ventilator-associated pneumonia (VAP)	Mortality, microbiological eradication, duration of mechanical ventilation, ICU and length of hospital stay, adverse drug events, and antimicrobial resistance.	Heterogeneity among studies	High
Alp E et al. (2006) [11]	Narrative review	Evidence-based strategies, including oral care and elevation of the head of the bed minimize VAP. Explains the CDC criteria for VAP.	ICU literature	Prevention of VAP	Review article	Low
Choi YH et al. (2021) [12]	Prospective observational study	Alterations in oral microbiota after endotracheal intubation and mechanical ventilation increased colonization by pathogenic bacteria that may contribute to VAP development.	Intubated mechanically ventilated patients	Oral colonisation with respiratory pathogens	Small observational study	Moderate
Ehrenzeller S et al. (2024) [13]	Systematic review and meta-analysis	Daily toothbrushing significantly reduced ventilator-associated pneumonia. Features the incorporation of toothbrushing into routine ICU oral care.	Hospitalised adults	Hospital-acquired pneumonia/VAP	Heterogeneity among included studies	High
Soh KL et al. (2012) [14]	Cross-sectional pilot survey	Demonstrated variation in oral care practices among ICU nurses, including frequency, techniques, and the usage of other oral care products like forceps, cotton, and gauze.	ICU nurses	Oral care practice variation	Survey	Low
Koeman M et al. (2006) [15]	Randomized controlled trial	Oral decontamination with chlorhexidine significantly reduced the incidence of ventilator-associated pneumonia compared with standard oral care.	Mechanically ventilated ICU patients	Ventilator-associated pneumonia (VAP) incidence	Single trial	High
Brookes ZLS et al. (2020) [16]	Narrative review	Reviewed clinical applications of chlorhexidine and potential adverse effects including tooth staining, taste disturbance, and mucosal irritation with prolonged use.	Human studies	Clinical efficacy and adverse effects	Narrative review	Low
Amano Y et al. (2020) [17]	Case report	Reported a life-threatening chlorhexidine-induced anaphylactic reaction during central venous catheter insertion.	Adult patient undergoing central venous catheter insertion	Chlorhexidine-induced anaphylaxis	Single-patient report	Very low
Emami Zeydi A et al. (2023) [18]	Systematic review and meta-analysis	Povidone-iodine oral care significantly reduced the incidence of ventilator-associated pneumonia compared with routine oral care.	Mechanically ventilated ICU patients	Ventilator-associated pneumonia	Heterogeneity among included trials	High
Tsuda S et al. (2020) [19]	Randomized controlled trial	Povidone-iodine significantly reduced oral bacterial colonization in mechanically ventilated patients and prevents ventilator-associated pneumonia.	Mechanically ventilated ICU patients	Oral bacterial colonisation and VAP prevention	Single-centre study	High
He Q et al. (2025) [20]	Network meta-analysis	Compared multiple oral mouthwashes used in ICU patients and studied their effectiveness in reducing ventilator-associated pneumonia.	ICU patients receiving mechanical ventilation	VAP and mortality	Heterogeneity of included trials	High
Newbrun E et al. (1996) [21]	Narrative review	Reviewed the role of sodium bicarbonate in oral hygiene. Concluded that sodium bicarbonate is a safe cleansing agent that neutralizes oral acidity and prevents the formation of dental plaque.	Dental health literature	Oral hygiene and plaque control	Narrative review	Low
Loha S et al. (2022) [22]	Prospective randomized controlled trial	Compared sodium bicarbonate mouthwash with standard oral care in traumatic brain injury patients. Sodium bicarbonate significantly reduced VAP incidence and improved oral hygiene.	Mechanically ventilated traumatic brain injury patients	Ventilator-associated pneumonia and oral hygiene	Single-centre	High
Koburger T et al. (2010) [23]	In vitro comparative laboratory study	Compared the antimicrobial efficacy of chlorhexidine, povidone-iodine, octenidine, polyhexanide, and triclosan. Octenidine and chlorhexidine demonstrated the strongest antimicrobial activity.	Laboratory (in vitro)	Antimicrobial efficacy	In vitro findings; no patient outcomes	Very low
Zumtobel M et al. (2009) [24]	Laboratory (in vitro) study	Octenidine-coated tracheostomy tubes significantly reduced bacterial colonization, suggesting a potential role in preventing respiratory infections.	Laboratory model using coated tracheostomy tubes	Bacterial colonisation	Laboratory model only	Very low
Bhargava HN (1996) [25]	Narrative review	Reviewed the antimicrobial applications and safety profile of triclosan. Reported broad-spectrum antibacterial activity.	Human and laboratory evidence	Antimicrobial efficacy and safety	Narrative review	Low
Sankaran SP (2020) [26]	Network meta-analysis	Compared oral care interventions for preventing VAP. Chlorhexidine and furacilin mouthwashes ranked among the most effective interventions.	Mechanically ventilated ICU patients	Prevention of ventilator-associated pneumonia	Heterogeneity and variable study quality	Moderate
Alshehri FA (2018) [27]	Systematic review	Reviewed essential oil-containing mouthwashes like Listerine. Found significant reductions in dental plaque and gingivitis, supporting antimicrobial effectiveness.	Adults with oral disease	Dental plaque and gingivitis	Dental population	Moderate
Li S et al. (2024) [28]	Systematic review and network meta-analysis	Evaluated miswak and other herbal oral care products for VAP prevention. Listerine reduced VAP incidence compared with conventional oral care.	Mechanically ventilated ICU patients	Ventilator-associated pneumonia	Heterogeneity and variable study quality	High
Vlachojannis C et al. (2016) [29]	Narrative review	Reviewed the efficacy and adverse effects of Listerine products. Demonstrated reductions in oral microbial load, plaque, and gingivitis.	Human oral health studies	Oral microbial load, plaque, and gingivitis	Narrative review	Low
de Paula GF et al. (2011) [30]	Laboratory (materials science) study	Explained the physical and chemical properties of polyhexamethylene biguanide (PHMB). Confirmed its stability and antimicrobial characteristics supporting its use as an antiseptic.	Laboratory	Physical and antimicrobial properties	Laboratory characterisation only; no clinical outcomes	Very low
Kollmuss M et al. (2021) [31]	In vitro biofilm study	Polyhexanide mouth rinse significantly reduced oral biofilm formation and inhibited the growth of oral pathogens, suggesting potential usefulness as an alternative oral antiseptic.	Laboratory biofilm model	Oral biofilm formation and pathogen growth	In vitro study; clinical efficacy unknown	Very low
Fernández-García C et al. (2016) [32]	Randomized clinical trial	Compared polyhexanide and povidone-iodine as oral care interventions in tracheotomised patients. Found improved oral hygiene with the usage of polyhexanide.	Tracheotomised ICU patients	Oral hygiene and tracheostomy care outcomes	Single-centre study	High
Vergara-Buenaventura A et al. (2020) [33]	Narrative review	Reviewed the antimicrobial activity of chlorhexidine, povidone-iodine, hydrogen peroxide, and cetylpyridinium chloride.	Dental literature	Antimicrobial activity of mouthwashes	Narrative review	Low
Nobahar M et al. (2016) [34]	Randomized controlled trial	Hydrogen peroxide mouthwash significantly reduced the incidence of ventilator-associated pneumonia compared with routine oral care and improved oral hygiene in critically ill patients.	Mechanically ventilated ICU patients	Ventilator-associated pneumonia and oral hygiene	Single-centre study	High
Walsh LJ (2000) [35]	Narrative review	Explained that low-concentration hydrogen peroxide is generally safe when appropriately used but excessive exposure may cause mucosal irritation.	Dental literature	Safety of hydrogen peroxide in oral care	Review	Low
Meidani M et al. (2018) [36]	Randomized controlled trial	Compared potassium permanganate with chlorhexidine for oropharyngeal irrigation. Both reduced VAP incidence, with chlorhexidine showing greater antimicrobial effectiveness.	Mechanically ventilated ICU patients	Ventilator-associated pneumonia	Single-centre study	High
Bansode P et al. (2024) [37]	Systematic review	Evaluated ozonated water for the management of gingivitis. Demonstrated antimicrobial and anti-inflammatory effects, supporting its potential use as an oral care measure.	Patients with gingivitis	Gingival inflammation and microbial control	Non-ICU population	Moderate
Izadi M et al. (2023) [38]	Double-blind randomized controlled trial	Ozonated water mouthwash significantly reduced ventilator-associated pneumonia compared with conventional oral care and improved oral microbial control.	Mechanically ventilated ICU patients	Ventilator-associated pneumonia and oral microbial control	Single-centre study	High
Ayala-Núñez NV et al. (2009) [39]	Laboratory (in vitro) study	Demonstrated potent bactericidal activity of silver nanoparticles against oral pathogens, supporting their potential antimicrobial application.	Laboratory	Bactericidal activity against oral pathogens	Laboratory study only; no clinical outcomes	Very low
Khaky B et al. (2018) [40]	Randomized clinical trial	Nanosil mouthwash significantly reduced pulmonary infections, including VAP, in ICU patients compared with standard oral care, suggesting it may be an effective alternative antiseptic.	Mechanically ventilated ICU patients	Pulmonary infection (including VAP)	Single-centre study	High
Dennesen P et al. (2003) [41]	Prospective observational study	Mechanically ventilated ICU patients developed reduced salivary flow, poor oral mucosal health, and increased dental plaque accumulation, emphasizing the importance of regular oral care during mechanical ventilation.	Mechanically ventilated ICU patients	Salivary flow, oral mucosal status and dental plaque	Observational design	Moderate
Barbe AG et al. (2017) [42]	Randomized, double-blind, crossover trial	Studied the efficacy of Biotène Oralbalance in medication-induced xerostomia. It improved oral moisture and xerostomia symptoms, supporting the importance of maintaining oral mucosal health.	Adults with medication-induced xerostomia	Xerostomia symptoms and oral moisture	Non-ICU population	Moderate
Stefanescu BM et al. (2013) [43]	Pilot clinical trial	Evaluated Biotène Oralbalance gel in mechanically ventilated preterm neonates. The gel was safe to use and improved oral care.	Mechanically ventilated preterm neonates	Oral health and safety	Pilot study; neonatal population	Low
Caruso P et al. (2009) [44]	Randomized controlled trial	Saline instillation before endotracheal suctioning significantly reduced the incidence of ventilator-associated pneumonia.	Mechanically ventilated ICU patients	Ventilator-associated pneumonia	Single-centre study	High
Caroff DA et al. (2016) [45]	Systematic review and meta-analysis	Subglottic secretion drainage significantly reduced ventilator-associated pneumonia, reduced the duration of mechanical ventilation, and delayed VAP onset.	Mechanically ventilated ICU patients	VAP incidence, duration of ventilation, time to VAP	Heterogeneity among included trials	High
Güner CK et al. (2021) [46]	Systematic review	Head-of-bed elevation (30°-45°) reduced the incidence of ventilator-associated pneumonia and should be incorporated into VAP prevention protocols.	Mechanically ventilated ICU patients	Ventilator-associated pneumonia	Variable quality of included studies	High
Shen KL et al. (2022) [47]	Randomized controlled trial	Artificial intelligence-assisted dental monitoring improved periodontal diagnosis compared with conventional examination, demonstrating the potential usage of AI.	Patients with periodontitis	Diagnostic accuracy for periodontal disease	Dental population	Moderate
Butnaru OM et al. (2025) [48]	Comparative diagnostic accuracy study	Artificial intelligence systems demonstrated diagnostic accuracy comparable to experienced clinicians for evaluating periodontal parameters when compared to general dentists.	Dental patients	Periodontal assessment accuracy	Non-ICU setting	Moderate
Unahalekhaka A et al. (2025) [49]	Multicentre prospective interventional study	Implementation of single-use oral care sets significantly reduced ventilator-associated pneumonia rates and improved adherence to standardized oral care practices in ICUs.	ICU patients receiving mechanical ventilation	Ventilator-associated pneumonia and protocol adherence	Non-randomised interventional design	Moderate
Richardson AK et al. (2025) [50]	Narrative review	Reviewed nanotechnology-based antimicrobial-coated endotracheal tubes. Concluded that antimicrobial coatings reduce bacterial colonization and may decrease VAP incidence.	Experimental and clinical literature	Prevention of bacterial colonisation and VAP	Narrative review	Low

Limitations

There are certain limitations of this review. This is a narrative review that aims to provide a concise overview of the available evidence regarding the interventions used for oral care in the ICU setting rather than functioning as a systematic review or meta-analysis. This review includes evidence from systematic reviews and randomised controlled trials (RCTs) conducted in relatively small patient populations and resource-limited settings that have not demonstrated statistically significant differences in the comparative studies, which may be insufficient to substantiate the effectiveness of that particular intervention. Several of the interventions discussed for oral care have been evaluated in only a limited number of studies to prove their efficacy on critically ill patients. Therefore, these interventions should be regarded as potential areas for future research rather than being adopted directly into clinical practice. An oral care regimen that encompasses the use of mouth rinses cannot always be administered to ventilated patients. This alteration in the route of administration may influence the efficacy of that intervention on critically ill patients. No universally accepted gold-standard route for delivering the oral care medication is mentioned in this review.

## Conclusions

It is understood that oral dysbiosis contributes significantly to the development of pneumonia in ventilated patients. However, there is still debate about the best ways to maintain good oral health in critical care settings. Oral hygiene strategies are effective in preventing VAP, but they also have some drawbacks.

New and promising drugs are being developed, but there is still a long way to go before these scientific advances are used in day-to-day practice. Clearer clinical guidelines and further research are needed to assess and effectively establish good oral hygiene practices in critically ill patients. This review aims to expand our knowledge regarding the available evidence for improving oral care in ICUs and preventing VAP.

## References

[1] Rello J, Diaz E (2003). Pneumonia in the intensive care unit. Crit Care Med.

[2] Torres A, Aznar R, Gatell JM (1990). Incidence, risk, and prognosis factors of nosocomial pneumonia in mechanically ventilated patients. Am Rev Respir Dis.

[3] Alp E, Güven M, Yildiz O, Aygen B, Voss A, Doganay M (2004). Incidence, risk factors and mortality of nosocomial pneumonia in intensive care units: a prospective study. Ann Clin Microbiol Antimicrob.

[4] (2025). Centers for Disease Control and Prevention. National Healthcare Safety Network (NHSN) Patient Safety Component Manual: Chapter 10—Ventilator-Associated Event (VAE). Atlanta, GA. CDC.

[5] Dong J, Li W, Wang Q, Chen J, Zu Y, Zhou X, Guo Q (2021). Relationships Between Oral Microecosystem and Respiratory Diseases. Front Mol Biosci.

[6] Rogers MB, Harner A, Buhay M (2024). The salivary microbiota of patients with acute lower respiratory tract infection-A multicenter cohort study. PLoS One.

[7] Liu X, Shi F, Zeng J (2025). Oral microbiota and respiratory diseases: advances and perspectives. Clin Microbiol Rev.

[8] Mehtonen IT, Rantala AK, Hugg TT, Jaakkola MS, Jaakkola JJ (2019). Dental caries is associated with lower respiratory tract infections: A population-based cohort study. Respir Med.

[9] Kollef MH (1993). Ventilator-associated pneumonia. A multivariate analysis. JAMA.

[10] Kalil AC, Metersky ML, Klompas M (2016). Management of Adults With Hospital-acquired and Ventilator-associated Pneumonia: 2016 Clinical Practice Guidelines by the Infectious Diseases Society of America and the American Thoracic Society. Clin Infect Dis.

[11] Alp E, Voss A (2006). Ventilator associated pneumonia and infection control. Ann Clin Microbiol Antimicrob.

[12] Choi YH, Kim SH, Yu Y (2021). Oral Microbiota Change in Intubated Patients under Mechanical Ventilation. J Bacteriol Virol.

[13] Ehrenzeller S, Klompas M (2024). Association Between Daily Toothbrushing and Hospital-Acquired Pneumonia: A Systematic Review and Meta-Analysis. JAMA Intern Med.

[14] Soh KL, Shariff Ghazali S, Soh KG, Abdul Raman R, Sharif Abdullah SS, Ong SL (2012). Oral care practice for the ventilated patients in intensive care units: a pilot survey. J Infect Dev Ctries.

[15] Koeman M, van der Ven AJ, Hak E (2006). Oral decontamination with chlorhexidine reduces the incidence of ventilator-associated pneumonia. Am J Respir Crit Care Med.

[16] Brookes ZL, Bescos R, Belfield LA, Ali K, Roberts A (2020). Current uses of chlorhexidine for management of oral disease: a narrative review. J Dent.

[17] Amano Y, Matsuura A, Tamura T, Kato Y, Kameyama N, Takazawa T, Nishiwaki K (2023). Life-threatening chlorhexidine anaphylaxis caused by skin preparation before chlorhexidine-free central venous catheter insertion: a case report and literature review. J Anesth.

[18] Emami Zeydi A, Parvizi A, Haddadi S (2023). Effect of Oral Care with Povidone-Iodine in the Prevention of Ventilator-Associated Pneumonia; a Systematic Review and Meta-Analysis. Arch Acad Emerg Med.

[19] Tsuda S, Soutome S, Hayashida S, Funahara M, Yanamoto S, Umeda M (2020). Topical povidone iodine inhibits bacterial growth in the oral cavity of patients on mechanical ventilation: a randomized controlled study. BMC Oral Health.

[20] He Q, Peng Z, He C, Zhang C, Hu R (2025). Effect of different mouthwashes on ventilator-related outcomes and mortality in intensive care unit patients: A network meta-analysis. Aust Crit Care.

[21] Newbrun E (1996). The use of sodium bicarbonate in oral hygiene products and practice. Compend Contin Educ Dent Suppl.

[22] Loha S, Kumar S, Reena Reena (2022). The effect of alkalinization of oral cavity by sodium bicarbonate mouth wash to decrease ventilator-associated pneumonia in traumatic brain injury patients: A prospective randomised controlled study. Trends in Anaesthesia and Critical Care.

[23] Koburger T, Hübner NO, Braun M, Siebert J, Kramer A (2010). Standardized comparison of antiseptic efficacy of triclosan, PVP-iodine, octenidine dihydrochloride, polyhexanide and chlorhexidine digluconate. J Antimicrob Chemother.

[24] Zumtobel M, Assadian O, Leonhard M, Stadler M, Schneider B (2009). The antimicrobial effect of Octenidine-dihydrochloride coated polymer tracheotomy tubes on Staphylococcus aureus and Pseudomonas aeruginosa colonisation. BMC Microbiol.

[25] Bhargava HN, Leonard PA (1996). Triclosan: applications and safety. Am J Infect Control.

[26] Sankaran SP, Sonis S (2021). Network meta-analysis from a pairwise meta-analysis design: to assess the comparative effectiveness of oral care interventions in preventing ventilator-associated pneumonia in critically ill patients. Clin Oral Investig.

[27] Alshehri FA (2018). The use of mouthwash containing essential oils (LISTERINE®) to improve oral health: A systematic review. Saudi Dent J.

[28] Li S, Huang Y, Xie H (2024). Herbal oral care products for the prevention of ventilator-associated pneumonia: A systematic review and network meta-analysis of randomised trials. PLoS One.

[29] Vlachojannis C, Al-Ahmad A, Hellwig E, Chrubasik S (2016). Listerine® Products: An Update on the Efficacy and Safety. Phytother Res.

[30] de Paula GF, Netto GI, Mattoso LHC (2011). Physical and chemical characterization of poly(hexamethylene biguanide) hydrochloride. Polymers.

[31] Kollmuss M, Tolksdorf K, Wuersching SN, Hickel R, Huth KC (2021). Effect of polyhexanide as antiseptic mouth rinse against oral pathogens in an in vitro biofilm model. Acta Odontol Scand.

[32] Fernández-García C, Alonso-Rodríguez A, Wensell-Fernández A (2016). [Randomised clinical trial to compare two tracheotomy care methods in an Intensive Care Unit]. Enferm Intensiva.

[33] Vergara-Buenaventura A, Castro-Ruiz C (2020). Use of mouthwashes against COVID-19 in dentistry. Br J Oral Maxillofac Surg.

[34] Nobahar M, Razavi MR, Malek F, Ghorbani R (2016). Effects of hydrogen peroxide mouthwash on preventing ventilator-associated pneumonia in patients admitted to the intensive care unit. Braz J Infect Dis.

[35] Walsh LJ (2000). Safety issues relating to the use of hydrogen peroxide in dentistry. Aust Dent J.

[36] Meidani M, Khorvash F, Abbasi S, Cheshmavar M, Tavakoli H (2018). Oropharyngeal Irrigation to Prevent Ventilator-Associated-Pneumonia: Comparing Potassium Permangenate with Chlorhexidine. Int J Prev Med.

[37] Bansode P, K S, D S, Muralidharan S (2024). Effectiveness of Ozonated Water on Gingivitis: A Systematic Review. Cureus.

[38] Izadi M, Bagheri M, Bahrami Far A, Bagheri-Baghdasht MS, Ghasemzadeh G, Sureda A, Soodmand M (2023). Reduce the risk of ventilator-associated pneumonia in ICU patients by Ozonated water mouthwash: A double-blind randomized clinical trial. Am J Infect Control.

[39] Ayala-Núñez NV, Villegas HHL, Turrent LdCI (2009). Silver nanoparticles toxicity and bactericidal effect against methicillin-resistant Staphylococcus aureus: nanoscale does matter. Nanobiotechnology.

[40] Khaky B, Yazdannik A, Mahjobipoor H (2018). Evaluating the Efficacy of Nanosil Mouthwash on the Preventing Pulmonary Infection in Intensive Care Unit: a Randomized Clinical Trial. Med Arch.

[41] Dennesen P, van der Ven A, Vlasveld M (2003). Inadequate salivary flow and poor oral mucosal status in intubated intensive care unit patients. Crit Care Med.

[42] Barbe AG, Schmidt-Park Y, Hamacher S, Derman SH, Noack MJ (2018). Efficacy of GUM® Hydral versus Biotène® Oralbalance mouthwashes plus gels on symptoms of medication-induced xerostomia: a randomized, double-blind, crossover study. Clin Oral Investig.

[43] Stefanescu BM, Hétu C, Slaughter JC, O'Shea TM, Shetty AK (2013). A pilot study of Biotene OralBalance® gel for oral care in mechanically ventilated preterm neonates. Contemp Clin Trials.

[44] Caruso P, Denari S, Ruiz SA, Demarzo SE, Deheinzelin D (2009). Saline instillation before tracheal suctioning decreases the incidence of ventilator-associated pneumonia. Crit Care Med.

[45] Caroff DA, Li L, Muscedere J, Klompas M (2016). Subglottic Secretion Drainage and Objective Outcomes: A Systematic Review and Meta-Analysis. Crit Care Med.

[46] Güner CK, Kutlutürkan S (2022). Role of head-of-bed elevation in preventing ventilator-associated pneumonia bed elevation and pneumonia. Nurs Crit Care.

[47] Shen KL, Huang CL, Lin YC (2022). Effects of artificial intelligence-assisted dental monitoring intervention in patients with periodontitis: A randomized controlled trial. J Clin Periodontol.

[48] Butnaru OM, Tatarciuc M, Luchian I (2025). AI Efficiency in Dentistry: Comparing Artificial Intelligence Systems with Human Practitioners in Assessing Several Periodontal Parameters. Medicina (Kaunas).

[49] Unahalekhaka A, Uirungroj P, Saenjum C (2025). Impact of single-use oral care sets on reducing ventilator-associated pneumonia among intensive care unit patients: a multi-centre study. J Hosp Infect.

[50] Richardson AK, Fuller RG, April MD (2026). Antimicrobial-coated endotracheal tubes: A narrative review. J Crit Care.

